# Constrictive pericarditis is an easily overlooked cause of right heart failure: a case report

**DOI:** 10.1186/1757-1626-1-27

**Published:** 2008-07-11

**Authors:** Aleksandra M Malkowska, W Stephen Waring

**Affiliations:** 1 Scottish Poisons Information Bureau, Royal Infirmary of Edinburgh, Edinburgh, UK

## Abstract

We describe a patient who suffered progressive right heart failure of unknown aetiology, despite a lengthy series of hospital investigations. Constrictive pericarditis had not been suspected during life, and was ultimately diagnosed as an autopsy finding. The salient clinical features and confirmatory investigations for this unusual disorder are reviewed. The case reminds us to consider the possibility of constrictive pericarditis in patients with unexplained chronic right heart failure, so that prompt investigation and treatment can be instigated.

## Case Report

A 76-year-old woman attended a gastroenterology outpatient clinic for investigation of unexplained weight loss and diffuse abdominal distension. Investigations showed deranged liver biochemistry tests, and computed tomography showed widespread ascites and a small pleural effusion but no focal organ pathology. Analysis of ascitic fluid showed total protein 34 g/L, and 3 white cells per mm^3^. The patient was subsequently referred to a cardiologist, who noted the presence of a soft systolic murmur. An echocardiogram showed normal left ventricular dimensions and function, but the right heart chambers were dilated and there was moderate to severe tricuspid regurgitation. *Cor pulmonale *was suspected and, therefore, the patient was referred to a respiratory clinic for investigation of possible underlying lung disease. The patient had been a non-smoker, with no past history of occupational exposure or tuberculosis. Spirometry was normal and computed tomography pulmonary angiography showed patent pulmonary arteries, and small bilateral pleural effusions. Cardiac catheterisation studies were proposed, but patient defaulted from clinic.

Eighteen months later, the patient was admitted to hospital in extremis. Initial examination found temperature 34.6°C, blood pressure 65/30 mmHg, and heart rate 85 min^-1^. The patient had reduced conscious level, cool peripheries, and elevated jugular venous pressure. An electrocardiogram showed atrial fibrillation and right bundle branch block. She was diagnosed with sepsis and dehydration, and treated with intravenous colloid administration, gradual warming, and systemic antibiotics. Despite these measures, hypotension and tissue perfusion progressively worsened and the patient died shortly after arrival in hospital.

Autopsy examination found extensive constrictive pericarditis and chronic hepatic congestion. Additional findings were colonic diverticular disease and gastrointestinal ischaemia. Death was attributed to heart failure secondary to constrictive pericarditis, exacerbated by sepsis and tissue ischaemia. The earlier investigative findings, which were performed over a 12-month duration, were attributable, in retrospect, to constrictive pericarditis. There had been no specific clinical signs suggestive of this diagnosis. The initial echocardiograph did not comment specifically on the pericardium, but is unlikely to have been sufficiently sensitive to detect pericardial thickening.

## Discussion

Constrictive pericarditis is a rare disorder that manifests predominantly as right heart failure and, in severe cases, systemic hypotension and circulatory collapse [1]. The diagnosis may be difficult to establish [2]. Histopathological features include localised fibrosis and calcification within the pericardium. All four cardiac chambers become encased so that diastolic pressure tend to equalise between them, which results in systemic venous engorgement and diminished ejection volume [3]. Ventricular filling during early diastole is rapid, due to high systemic venous pressure, whereas late diastolic filling is impaired by the rigid pericardium. Cardiac output may be preserved, at least in part, due to high diastolic filling pressure. A comparatively small reduction in circulating volume and central venous pressure can cause a dramatic fall in ejection volume and cardiac output.

Constrictive pericarditis may arise as a complication of previous cardiac surgery, pericarditis, and mediastinal irradiation [4]. Other recognised but rare causes include connective tissue disorders, malignancy, and local trauma. Tuberculosis is uncommon in Western nations, although occurs more commonly in developing countries. In around one third, no underlying cause is identified, although adenovirus or echovirus infection has been implicated [5]. Clinical signs include oedema, ascites, raised jugular venous pressure, pleural effusion, and hepatomegaly. Around one third of patients with constrictive pericarditis have co-existent atrial fibrillation [6]. Plain chest radiography may show pericardial calcification and unexplained pleural effusions [2]. Echocardiography may confirm the presence of small ventricular dimensions with preserved systolic function, and dilated atria. Abrupt termination of diastolic filling may cause a characteristic 'septal bounce' [7]. Cardiac catheterisation studies allow definitive confirmation. Constrictive pericarditis may be distinguished from restrictive cardiomyopathy by demonstration of thickened pericardium on computed tomography and magnetic resonance imaging [8].

Surgical intervention may be appropriate, depending on the severity of haemodynamic impairment. Total rather than partial pericardiectomy yields better outcome, and should be undertaken early, where possible [9]. Survival is dependent on the underlying aetiology, and patients with idiopathic constrictive pericarditis tend to have best outcome after pericardiectomy [10].

## Conclusion

Right heart failure may be caused by a variety of disorders, including *cor pulmonale*, right ventricular infarction, mitral stenosis, pulmonary embolism, and primary pulmonary hypertension. Constrictive pericarditis is a comparatively rare cause, and the diagnosis may be difficult to establish. This case reminds us to consider constrictive pericarditis in patients with unexplained right heart failure, so that timely investigation and treatment can be initiated.

## Consent

Written informed consent was obtained from the patient's next of kin for publication of this case report. A copy of the written consent is available for review by the Editor-in-Chief of this journal.

## Competing interests

The authors declare that they have no competing interests.

## Authors' contributions

AMM performed a literature review and prepared a draft manuscript; WSW contributed to manuscript writing and editing. Both authors read and approved the final manuscript.
